# Phthisis in a Monkey

**Published:** 1881-01

**Authors:** 


### VI.—PHTHISIS IN A MONKEY (*Macacus pileatus*), CENTRAL PARK MENAGERIE.

NECROPSY.—The body was somewhat emaciated. Other external peculiarities were not marked. The abdominal cavity contained no fluid; the bladder was distended with clear urine; the spleen was greatly enlarged, and contained many large variously-shaped cavities, showing separate membranes, and filled with creamy or cheesy masses. Stomach, liver, and intestines presented no abnormality. Both kidneys were large, had non-adherent capsules, but showed numerous small yellowish or grey spots disseminated through their substance. On opening the thorax, the left lung was found to be healthy, but on the right side there was no remnant of normal pulmonary tissue. The entire pleural cavity of that side was filled up with a cheesy, crumbling mass, which adhered to the pericardium and had encroached on the heart. In this yellow substance rounded or oval encapsulated accumulations, of a firmer consistence than the surrounding cheesy mass, were discovered. Two of these—one about the size of a cherry, the other as large as a pea—were firmly attached to the costal pleura. The substernal and bronchial glands were likewise in a condition of uniform cheesy degeneration. The heart showed moderate hypertrophy, especially of its left ventricle. On opening the skull, the dura mater was seen to be somewhat thickened, principally at the convexity. The pia mater showed numerous variously sized ecchymoses, but was not opaque or thickened. The brain itself appeared normal. E. C. W.

